# TGFβ Pathway Inhibition Redifferentiates Human Pancreatic Islet β Cells Expanded *In Vitro*

**DOI:** 10.1371/journal.pone.0139168

**Published:** 2015-09-29

**Authors:** Ginat Toren-Haritan, Shimon Efrat

**Affiliations:** Department of Human Molecular Genetics and Biochemistry, Sackler School of Medicine, Tel Aviv University, Tel Aviv, Israel; University of Torino, ITALY

## Abstract

*In-vitro* expansion of insulin-producing cells from adult human pancreatic islets could provide an abundant cell source for diabetes therapy. However, proliferation of β-cell-derived (BCD) cells is associated with loss of phenotype and epithelial-mesenchymal transition (EMT). Nevertheless, BCD cells maintain open chromatin structure at β-cell genes, suggesting that they could be readily redifferentiated. The transforming growth factor β (TGFβ) pathway has been implicated in EMT in a range of cell types. Here we show that human islet cell expansion *in vitro* involves upregulation of the TGFβ pathway. Blocking TGFβ pathway activation using short hairpin RNA (shRNA) against *TGFβ Receptor 1* (*TGFBR1*, *ALK5)* transcripts inhibits BCD cell proliferation and dedifferentiation. Treatment of expanded BCD cells with *ALK5* shRNA results in their redifferentiation, as judged by expression of β-cell genes and decreased cell proliferation. These effects, which are reproducible in cells from multiple human donors, are mediated, at least in part, by AKT-FOXO1 signaling. *ALK5* inhibition synergizes with a soluble factor cocktail to promote BCD cell redifferentiation. The combined treatment may offer a therapeutically applicable way for generating an abundant source of functional insulin-producing cells following *ex-vivo* expansion.

## Introduction

β-cell replacement by transplantation is a promising treatment for type 1 diabetes, however it is limited by the shortage of human donors. *In-vitro* expansion of adult human islet cells from cadaver donors may increase β-cell availability, however β cells rapidly lose their phenotype when induced to proliferate in culture [1]. Using a lentivirus-based lineage tracing system we have tracked β-cell derived (BCD) cell fate in culture [2] and associated the phenotypic changes with epithelial-mesenchymal transition (EMT) [3]. EMT is a cellular process involved in development, tissue repair, and disease, and is characterized by loss of epithelial markers, such as E-cadherin, and gain of mesenchymal markers, including N-cadherin and α-smooth muscle actin (α-SMA) [4]. EMT is induced by several signaling pathways, including WNT, NOTCH, Hedgehog, and transforming growth factor β (TGFβ) [5, 6]. TGFβ signals are conveyed through two transmembrane serine-threonine kinase receptors (type I and type II TGFβ receptors) to the five receptor-regulated (R)-SMAD transcription factors (SMAD1-3, 5, 8), which translocate into the nucleus, recruit transcriptional co-activators and co-repressors, and regulate gene expression [7]. TGFβ signaling involves over 60 components, which interact in numerous combinations to fine-tune multiple cellular processes [8–10]. The type I receptor family is comprised of activin-like kinase (ALK) receptors 1 through 7. Two major pathway branches are activated by TGFβ family ligands: SMAD1, 5 and 8 are activated by BMPs through ALK1-3 and 6, whereas SMAD2 and 3 are activated by TGFβ, activins, and nodals, through ALK4, 5 and 7 [11, 12]. SMAD2 and 3 phosphorylation by ALK5 (also termed TGBRI) is the best-characterized TGFβ pathway signaling effect [13], and the main one associated with EMT, whereas mesenchymal-epithelial transition (MET) is associated primarily with phosphorylation of SMAD1, 5 and 8 [6, 14–17]. The ALK5/SMAD2 and 3-dependent pathway branch constitutes a major therapeutic target in cancer [18–20]. In addition to the pivotal role of the SMAD-dependent canonical TGFβ pathway in EMT, emerging evidence suggests that non-canonical TGFβ signaling, which activates GTPases, MAP kinases, and the growth and survival promoting kinases PI3K, AKT/PKB and mTOR, plays key roles in many cellular processes, including EMT [21–23].

We have previously reported that BCD cells maintain an open chromatin structure at β-cell genes [24] and can be redifferentiated by a combination of soluble factors [25] and by inhibition of the NOTCH pathway [26]. Redifferentiation leads to partial restoration of the β-cell phenotype in a fraction of BCD cells. Here we evaluated the effect of TGFβ pathway downregulation on redifferentiation of β cells expanded *in vitro*.

## Methods

### Ethics statement

This study was conducted according to the principles expressed in the Declaration of Helsinki. The Institutional Review Boards of the following medical centers, which provided human islets, each provided approval for the collection of samples and subsequent analysis: University of Geneva School of Medicine; San Raffaele Hospital, Milan; Faculty of Medicine, Lille 2 University; Massachusetts General Hospital; Washington University; University of Pennsylvania; Scharp/Lacy Institute; University of Illinois; University of Wisconsin; University of Miami; Southern California Islet Consortium. All donors provided written consent for the collection of all samples and subsequent analysis.

### Cell culture and redifferentiation

Human islets (Table 1) were received 2–6 days following isolation. Islets were dissociated into single cells. Cells were cultured as previously described [1] in CMRL 1066 medium containing 5.6 mM glucose and supplemented with 10% fetal bovine serum (FBS) (HyClone, Logan, UT); 100 units/ml penicillin, 100 mg/ml streptomycin, and 100 mg/ml gentamycin (PSG); 5 mg/ml amphotericin B; and 3.5 mg/ml L-glutamine. The cells were refed twice a week and split 1:2 once a week. 293T cells were cultured in DMEM supplemented with 10% FBS, PSG, and 3.5 mg/ml L-glutamine. Redifferentiation cocktail (RC), consisting of 1% BSA fraction V (Sigma), 1X insulin/transferrin/selenium (ITS, Invitrogen), D-Glucose (final concentration 25 mM), 8 nM exendin-4 (Acris), 8 nM activin A (Cytolab/PreproTech), 1X B27 supplement (Stem Cell Technologies), and 10 mM nicotinamide (Sigma), in CMRL 1066 medium supplemented with PSG, was prepared and applied to cells as previously described [27]. ALK5 inhibitor II (Enzo), and FOXO1 inhibitor AS1842856 (Millipore), were applied to cells every 48 hours at a final concentration of 0.1 μM.

**Table 1:** Donors of islets used in the study.

Donor no.	Donor gender	Donor age (Y)	Donor BMI	Islet purity (%)	Cell viability (%)
1	M	40	29	95	NA
2	F	62	27	95	71
3	F	13	24	88	65
4	F	66	24	82	58
5	F	58	27	99	70
6	F	54	29	83	73
7	M	46	24	74	70
8	F	45	26	90	66
9	M	41	22	70	NA
10	F	32	27	80	78
11	F	44	22	85	52
12	F	63	23	70	76
13	M	34	28	85	81
14	F	46	33	80	68
15	M	59	25	85	43
16	M	52	21	80	50
17	M	29	26	70	NA
18	M	34	28	90	71
19	F	44	35	95	76
20	M	48	18	90	77
21	M	49	31	90	65
22	M	45	27	85	74
23	F	20	25	85	43
24	F	51	21	85	65
25	F	41	36	90	65
26	F	47	21	90	NA
27	M	31	29	85	59
28	F	60	35	80	75
29	F	32	29	80	44
30	F	44	33	90	79
31	F	48	37	90	58
32	F	51	29	88	40
33	M	62	28	80	73
34	F	49	37	94	63
35	F	47	30	90	57
36	F	61	31	90	70
37	F	45	34	80	76
38	F	29	21	90	80
39	F	48	33	90	67
40	M	48	31	80	79
41	M	15	23	90	67
42	F	47	33	70	NA
43	F	27	23	70	NA
44	M	21	34	85	71
45	M	44	25	99	80
46	M	27	19	85	57
47	M	39	27	98	66
48	M	42	35	92	82
49	M	38	30	85	71
50	M	29	30	95	88
51	M	42	33	93	82
52	F	49	27	90	63
53	M	54	33	85	NA
54	M	62	19	92	69
55	M	50	28	85	70
56	M	47	33	90	70
57	F	48	22	95	78
Mean±SD		44±12	28±5	86±7	68±8

### Virus production, cell infection and cell sorting

Lentiviral vectors encoding ALK5 shRNAs (accession numbers TRCN-4693, -6309, -6326,-9773, and 9777), AKT1 shRNAs (accession numbers TRCN-0162, -0163, -0174, -9794, and -9797), and a non-target shRNA, in plko.1-PURO, were obtained from the RNAi Consortium (Sigma-Aldrich). Lineage tracing was performed using the RIP-Cre/ER and pTrip–loxP-NEO-STOP-loxP-eGFP lentiviral vectors as previously described [3]. Virus was produced in 293T cells as previously described [26]. Cells were infected at MOI 2:1 in CMRL 1066 medium containing 8 mg/ml polybrene overnight. The medium was then replaced with culture medium. Four days following infection the cells were selected with 1 mg/ml puromycin for 3 days. eGFP-labeled cells were sorted using a FACS Aria sorter as described [2].

### qPCR analysis

Total RNA was extracted using the ZR RNA MiniPrep RNA Isolation Kit (Zymo), and treated with RNase-free DNase I (Thermo). cDNA was produced using High-Capacity cDNA Reverse Transcription Kit (Applied Biosystems). qPCR was carried out in triplicates using the Universal Probe Library System (Roche) in 7300 Real-time PCR system (Applied Biosystems). Results were normalized to the TATA-box-binding protein (TBP) or Ribosomal protein large P0 (RPLP0) transcripts. Data analysis was performed with qBase software. S1 Table lists primer sequences. All reactions were performed with annealing at 60°C for 40 cycles. For undetectable transcripts, the cycle number was set to 40 for comparisons.

### Immunoblotting analysis

Cellular protein was extracted for 10 min in 50 mM Tris-HCl buffer, pH 7.4, containing 0.5% NP-40, 0.7% NaCl, 0.2% EDTA, and protease inhibitor cocktail. Samples of 20–30 μg protein were resolved by SDS-PAGE and transferred to PVDF membrane using Trans-Blot® Turbo™ RTA Transfer kit (Bio-Rad). Non-specific sites were blocked for one hour at room temperature (RT) in blocking buffer containing 5% skim milk, or 5% BSA, in TTBS buffer. The membrane was then incubated with primary antibody (S2 Table) diluted in blocking buffer. The bound antibody was visualized with the corresponding horseradish peroxidase-conjugated anti-IgG (Jackson) and SuperSignal West Chemiluminescent Substrate kit (Pierce). Signal intensity was quantitated using TINA software.

### Immunoflourescence analysis

Cells were spotted on slides using Shandon Cytospin4 centrifuge (Thermo Scientific), and fixed for 10 minutes at RT in 4% paraformaldehyde. For nuclear antigens, cells were incubated in Methanol for 5 minutes at -20°C prior to blocking. Samples were blocked for 30 min at RT in blocking buffer (1% BSA, 10% fetal goat serum, and 0.2% saponin) and incubated overnight at 4°C, or 1 hour at RT, with primary antibodies (S2 Table) diluted in blocking buffer. Slides were washed three times in TTBS, and incubated with the corresponding secondary antibody conjugated to Alexa fluorophores (1:1000, all from Invitrogen). DNA was stained with DAPI. The slides were mounted with Fluorescent Mounting Medium (GBI Labs). Images were visualized under a fluorescent BX61 microscope or TCS SP5 confocal fluorescent microscope (Leica). To demonstrate antibody specificity, a minus-primary antibody control was employed.

### Apoptosis detection assay

TUNEL assay was performed using In Situ Cell Death Detection Kit (Roche), according to the manufacturer’s protocol. DNase I-treated specimen served as a positive control, according to manufacturer’s protocol. The fluorescence was visualized under a fluorescent BX61 microscope.

### Insulin content and secretion

Cells were pre-incubated for one hour in Krebs–Ringer buffer (KRB), followed by incubation for two hours in KRB containing 0.5 mM 1-isobutyl 3-methylxanthine (IBMX) and 16.7 mM glucose. C-peptide content was determined in acidic alcohol cell extract. Human C-peptide was quantified using an ultrasensitive ELISA kit (Mercodia, Uppsala, Sweden; sensitivity 1.5 pmol/L; cross-reactivity with insulin and proinsulin 0.0006% and 1.8%, respectively) according to the manufacturer’s protocol.

### cDNA microarray analysis

Hybridization to Affymetrix GeneChip Human Gene 1.0 ST Arrays, washing, and scanning, were performed according to the manufacturer. Data analysis was performed on CEL files using Partek Genomics Suite software (Partek). Data were normalized with the multi-average method. Batch effect removal was applied for the different samples, followed by one-way ANOVA. Clustering analysis was performed by Partek Genomics Suite software with Pearson’s dissimilarity correlation by average linkage methods. The raw data has been deposited in the GEO database (accession number GSE60803).

### Statistical analysis

Significance was determined using two-tailed t-test. To approach a normal distribution, a logarithmic transformation was performed. To account for multiple testing, the Bonferroni correction was applied.

## Results

### TGFβ pathway activation in islet cell culture

**Fig 1:**
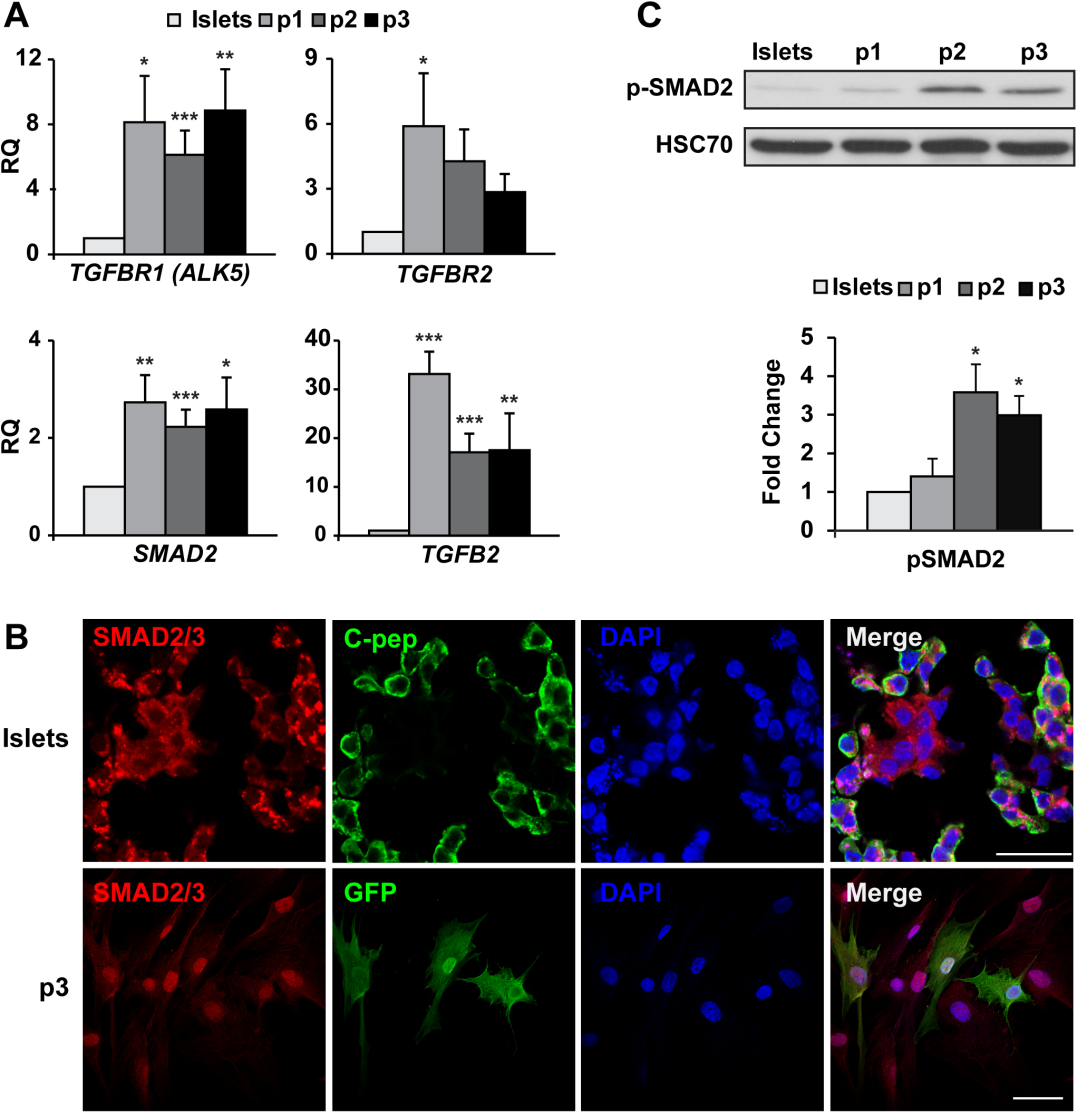
TGFβ pathway is activated in expanded human islet cells. A, qPCR analysis of RNA extracted from isolated human islets and expanded islet cells at the indicated passages. Values are mean±SE (n = 6 donors) relative to islets (RQ = 1) and normalized to *RPLPO* and *TBP*. *P≤0.05, **P≤0.01, ***P≤0.001. B, Immunofluorescence analysis of SMAD2/3 in human islets and expanded islet cells at passage 3. SMAD2/3 is localized in the cytoplasm of β-cells, and in the nucleus of GFP^+^ BCD cells. Bar = 25 μm (top), 50 μM (bottom). DNA was stained with DAPI. 100% of GFP^+^ cells showed staining of SMAD2/3 in the nucleus, based on counting ≥500 cells in each of 3 samples from different donors. C, Immunoblotting analysis of phosphorylated SMAD2 in human islets and expanded islet cells at the indicated passages. Values are mean±SE (n = 4–5 donors) relative to islets and normalized to HSC70. *P≤ 0.05.

Analysis of changes in transcripts encoding TGFβ pathway components during the first three weeks of islet cell culture revealed a significant upregulation of *TGFBR1* transcripts, as well as those encoding SMAD2 and TGFB2 (Fig 1A). A shift in SMAD2/3 localization from the cytoplasm in C-peptide^+^ cells to the nucleus in GFP^+^ BCD cells during this period (Fig 1B) supports the activation of the TGFβ pathway in these cells. Immunoblotting analysis further supported the finding of SMAD2 activation by revealing a 3.5-fold increase in SMAD2 phosphorylation during the first three weeks of islet cell culture (Fig 1C). In contrast to the changes in SMAD2/3 expression and localization, qPCR analysis of *SMAD1*, *5*, *8*, and *BMP7*, and immunofluorescence analysis of SMAD1/5/8 did not show activation of this branch of the TGFβ pathway (S1 Fig). Taken together, these findings suggest an activation of the TGFβ pathway branch reported to be responsible for EMT rather then MET.

**Fig 2:**
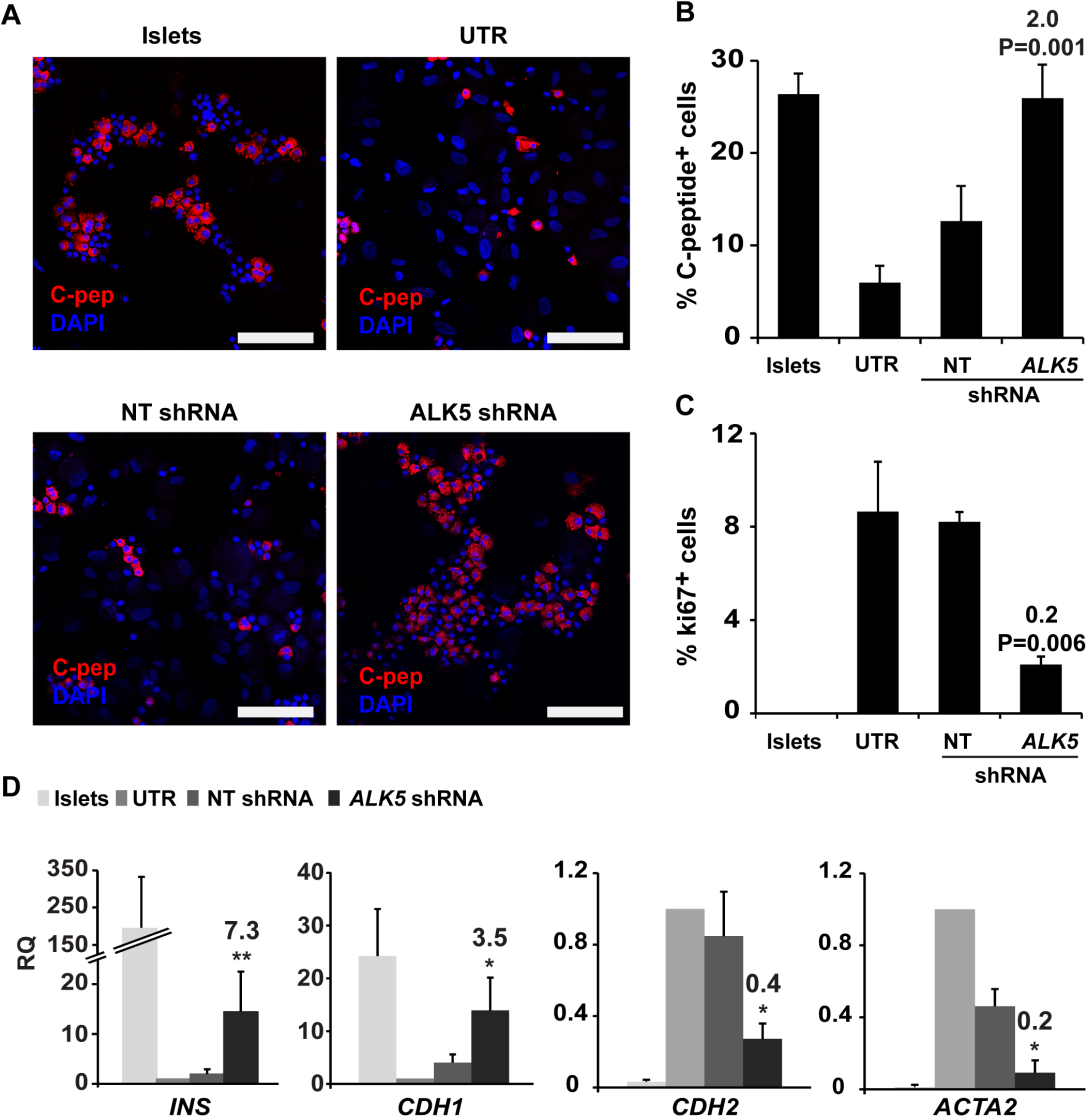
Blocking of ALK5 activation prevents proliferation and dedifferentiation in cultured islet cells. A,B, Immunofluorescence analysis of C-peptide expression in human islet cells infected with *ALK5* or nontarget (NT) shRNA viruses and grown in culture for 3 weeks, compared with uncultured islets and untreated (UTR) cultured cells. Bar = 100 μM. DNA was stained with DAPI. C, Quantitation of immunofluorescence analysis of Ki67 expression in the cells in A. Values in B and C are mean±SE (n = 4–5 donors), based on counting ≥500 cells in each sample. Fold change and P values shown on top of bars are relative to NT shRNA. D, qPCR analysis of RNA extracted from the cells in A. Values are mean±SE (n = 4–5 donors) relative to UTR (RQ = 1) and normalized to *RPLPO* and *TBP*. *P≤ 0.05, **P≤0.01. Fold change and P values shown on top of bars are relative to NT shRNA.

To determine the effects of blocking the TGFβ pathway, ALK5 expression was inhibited using shRNA. Five *ALK5* shRNAs were evaluated for their effects on p-SMAD2 and *INS* transcript levels in expanded islet cells (S2 Fig). Based on these analyses, shRNA 9 (TRCN-6309) was chosen for further detailed analyses, and will be referred to as *ALK5* shRNA. This shRNA reduced *ALK5* transcript levels by 70% (S2 Fig), and ALK5 protein by 40% (S2 Fig). The *ALK5* shRNA treatment did not result in a detectable increase in apoptosis, compared with nontarget (NT) shRNA (S3 Fig). Blocking ALK5 upregulation during the first 3 weeks of islet cell culture using *ALK5* shRNA prevented dedifferentiation, as judged by the percent of C-peptide^+^ cells in *ALK5* shRNA-treated cells, compared to controls (Fig 2A and 2B). In addition, *ALK5* shRNA partially blocked induction of cell proliferation, as judged by Ki67 expression (Fig 2C). Although *INS* and *CDH1* transcript levels decreased in *ALK5* shRNA-treated cells, compared with uncultured islets, they were significantly higher, compared to cells treated with NT shRNA, while transcripts encoding the mesenchymal markers CDH2 and ACTA2 were significantly less elevated (Fig 2D). Taken together, these findings indicate that blocking TGFβ pathway activation in cultured human islet cells prevents the induction of cell proliferation, and partially blocks cell dedifferentiation and EMT.

### Effect of ALK5 inhibition on BCD cell redifferentiation

**Fig 3:**
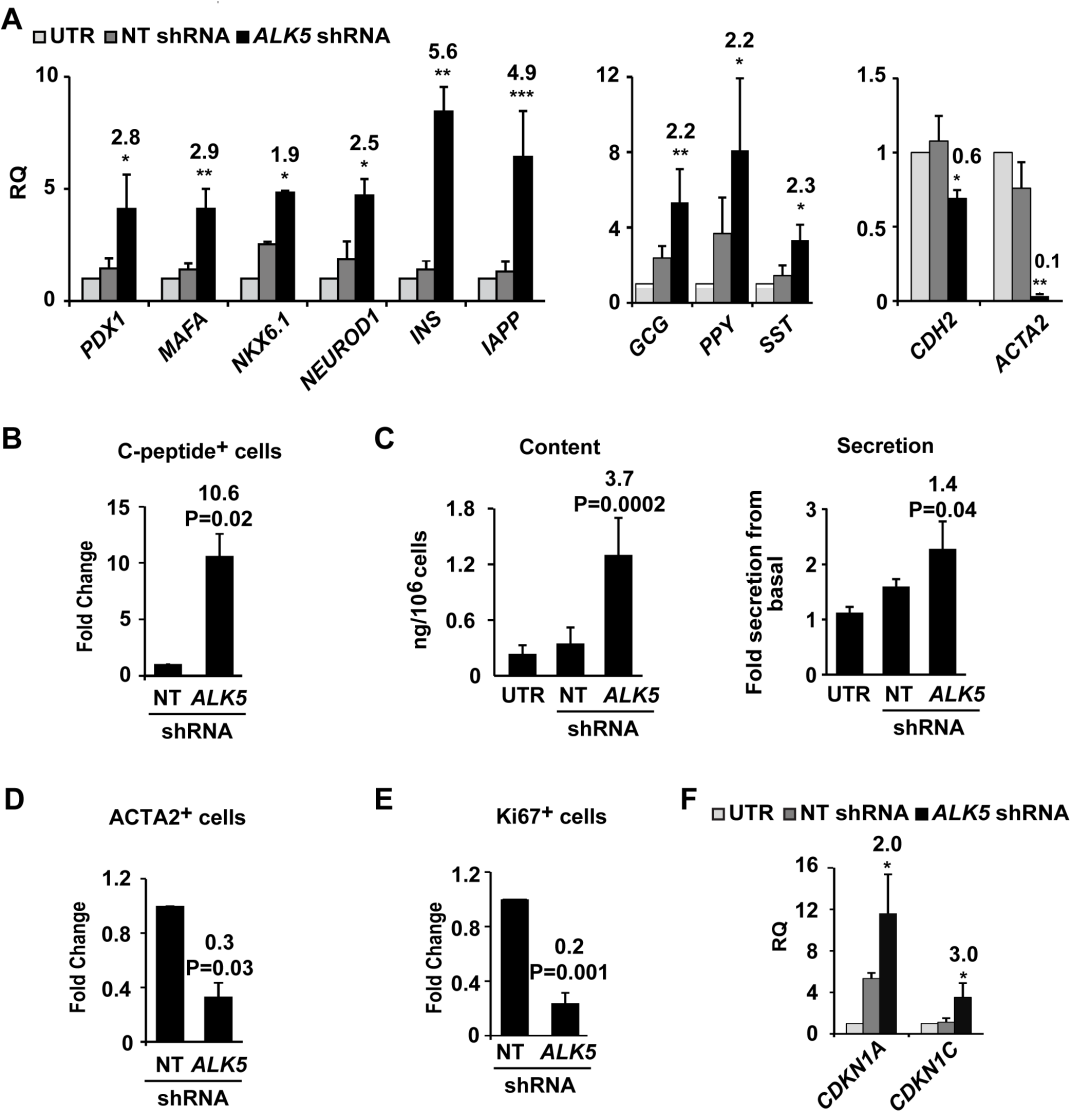
Effect of ALK5 inhibition on redifferentiation of expanded human islet cells. Cells were infected at passage 5 with *ALK5* or NT shRNA viruses and analyzed 6 days later. A, qPCR analysis of transcripts encoding β-cell proteins (left), pancreatic hormones (middle), and mesenchymal markers (right).Values are mean±SE (n = 3–8 donors), relative to UTR and normalized to *RPLPO* and *TBP*. *P≤0.05, **P≤0.01, ***P≤0.001. B, Quantitation of immunofluorescence analysis of C-peptide. Values are mean±SE (n = 3 donors), based on counting ≥500 cells in each sample. C, C-peptide content and glucose-induced secretion. Values are mean±SE (n = 4 donors). D, E, Quantitation of immunofluorescence analysis of ACTA2 and Ki67. Values are mean±SE (n = 3 donors), based on counting ≥500 cells in each sample. F, qPCR analysis of transcripts encoding cell cycle inhibitors. Values are mean±SE (n = 3–5 donors) relative to UTR and normalized to *RPLPO* and *TBP*. *P≤0.05. Fold change and P values shown on top of bars are relative to NT shRNA.

To evaluate the effects of TGFβ pathway inhibition on reversal of BCD cell dedifferentiation, expanded islet cells were treated with *ALK5* shRNA. qPCR analysis revealed a significant upregulation of transcripts encoding insulin, IAPP, and β-cell transcription factors, relative to cells treated with control NT shRNA (Fig 3A left), as well as an upregulation of transcripts of other pancreatic islet hormones (Fig 3A middle), while *CDH2* and *ACTA2* transcripts were downregulated (Fig 3A right), suggesting the induction of MET. Inhibition of ALK5 using a small molecule inhibitor, ALK5 inhibitor II, resulted in insulin transcript induction (S4 Fig); however, this effect was significantly lower, compared to *ALK5* shRNA-treated cells. The number of C-peptide^+^ cells among cells treated with *ALK5* shRNA increased 10.6-fold, compared to cells treated with NT shRNA (Fig 3B). C-peptide content of *ALK5* shRNA-treated cells, 1.3±0.4 ng/10^6^ cells, was 3.7-fold higher than that of NT shRNA-treated cells (Fig 3C, left). *ALK5* shRNA-treated cells responded to glucose with a 2.3-fold increase in C-peptide release (Fig 3C, right), compared to a 3-fold increase observed in islets [27]. A 3.3-fold decrease in ACTA2-expressing cells in *ALK5* shRNA-treated cells, relative to the control shRNA (Fig 3D), along with a 5-fold decrease in the number of Ki67-positive cells (Fig 3E), indicates reversal of EMT and induction of growth arrest. These findings were supported by upregulation of *CDKN1A* and *CDKN1C* transcripts, encoding the cell cycle inhibitors p21 and p57, respectively, in *ALK5* shRNA-treated cells, compared to controls (Fig 3F).

**Fig 4:**
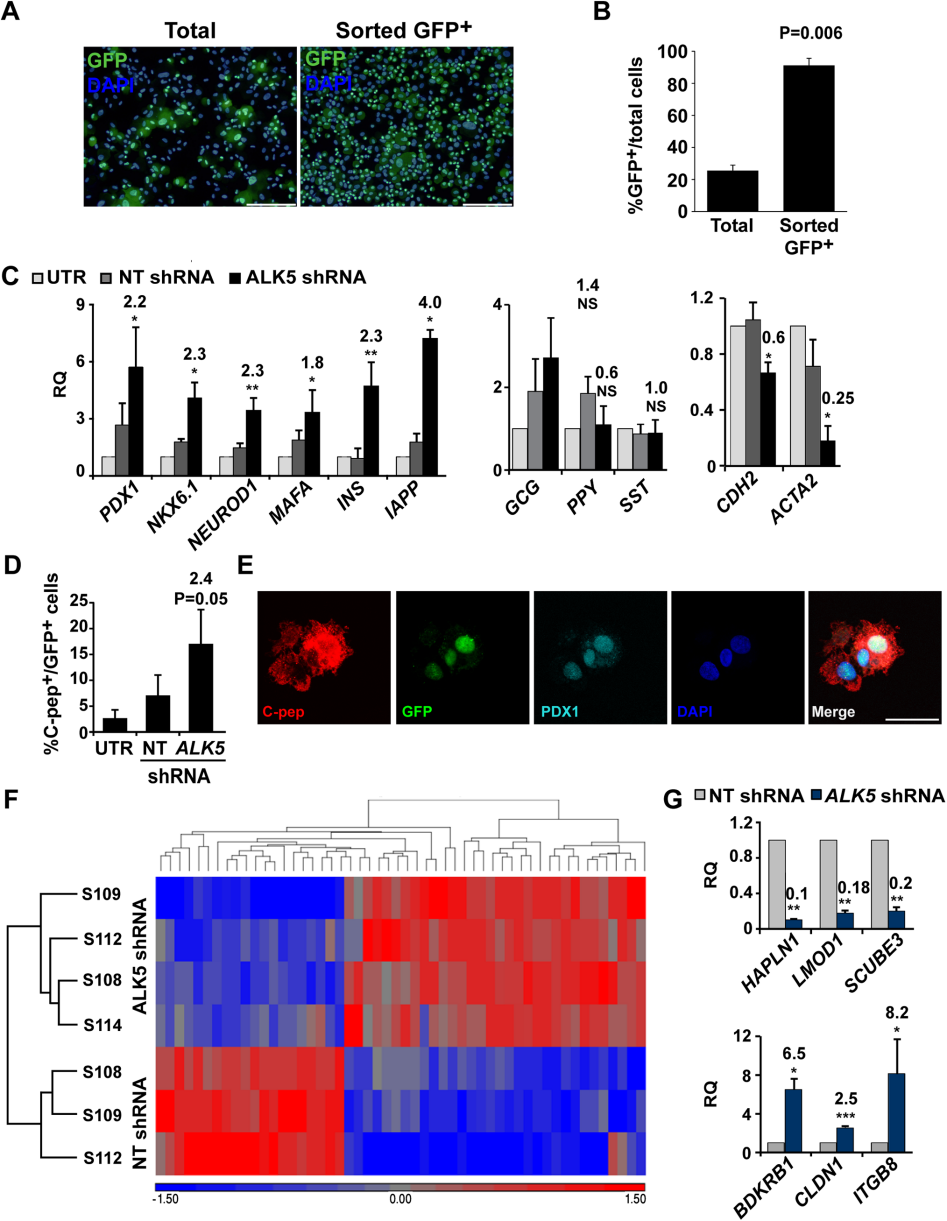
Effect of ALK5 inhibition on redifferentiation of BCD cells. A, Immunofluorescence analysis of GFP in expanded islet cells at passages 2–3, compared to sorted GFP^+^ cells. Bar = 200 μM. DNA was stained with DAPI. B. Quantitation of cells in A, based on counting ≥500 cells in each of 3 samples from different donors. C, qPCR analysis of transcripts encoding β-cell proteins (left), pancreatic hormones (middle), and mesenchymal markers (right), in RNA extracted from sorted GFP^+^ BCD cells infected at passage 5 with *ALK5* or NT shRNA viruses, and analyzed 6 days later. Values are mean±SE (n = 3–6 donors) relative to UTR and normalized to *RPLPO* and *TBP*. *P≤ 0.05, **P≤0.01. Fold change and P values shown on top of bars are relative to NT shRNA. D, Quantitation of immunofluorescence analysis of C-peptide and GFP in cells infected at passage 5 with *ALK5* or NT shRNA viruses and analyzed 6 days later. Values are mean±SE (n = 4 donors), based on counting ≥500 cells in each sample. Fold change and P value are relative to NT shRNA. E, Co-expression of C-peptide and PDX1 in GFP^+^ BCD cells infected at passage 5 with *ALK5* or NT shRNA viruses and analyzed 6 days later by immunofluorescence. Bar = 25 μM. DNA was stained with DAPI. F, Hierarchical clustering of 32 upregulated and 20 downregulated (>1.5 fold, pV≤0.05) transcripts in cDNA microarray analyses of RNA extracted from sorted BCD cells infected at passages 4–5 with *ALK5* (n = 4 donors) or NT (n = 3 donors) shRNA viruses and analyzed 6 days later. G, qPCR validation of cDNA microarray results for selected genes. Values are mean±SE (n = 3–7 donors) relative to NT shRNA and normalized to *RPLPO* and *TBP*. *P≤0.05, **P≤0.01, ***P≤0.001. Fold change and P values shown on top of bars are relative to NT shRNA.

The effects of *ALK5* shRNA in expanded islet cells were reproduced in sorted BCD cells (purity 91%±4%; Fig 4A and 4B). Levels of transcripts encoding insulin, IAPP, and β-cell transcription factors were significantly upregulated, compared to NT shRNA control (Fig 4C left), suggesting that the bulk of upregulation in these transcripts observed in the expanded mixed islet cell population represented BCD cell redifferentiation. In contrast, transcripts encoding other pancreatic hormones did not show a significant change in expression (Fig 4A middle), suggesting that the changes in levels of these transcripts observed in the expanded mixed islet cell population were not due to their upregulation in BCD cells. As seen in the expanded mixed islet cell population, *CDH2* and *ACTA2* transcripts were significantly downregulated by ALK5 shRNA treatment (Fig 4C, right), suggesting the induction of MET in BCD cells. In accordance with the RNA data, immunostaining revealed a 2.4-fold increase in the number of C-peptide^+^ cells in BCD cells (Fig 4D and 4E). Virtually all C-peptide^+^ cells co-stained for PDX1. To assess global changes in gene expression following downregulation of ALK5, RNA extracted from sorted BCD cells treated with *ALK5* or NT shRNA was subjected to cDNA microarray analysis. The analysis revealed that 32 transcripts were upregulated >1.5 fold (pV≤0.05), including those encoding the β-cell marker IAPP, while 20 transcripts were downregulated >1.5 fold (pV≤0.05), including *ACTA2* (Fig 4F). DAVID functional annotation revealed that these genes shared the terms Integral to plasma membrane (P = 0.001) and Cell adhesion (P = 0.02), consistent with induction of MET. qPCR analysis of selected genes (Table 2) confirmed the cDNA microarray results (Fig 4G). Among downregulated genes were *HAPLN1*, involved in cell adhesion, *LMOD1*, responsible for actin binding, and *SCUBE3*, that has been reported to positively regulate EMT via the TGFβ pathway [28]. Upregulated genes included *CLDN1*, encoding a tight-junction component, ITGB8, a member of the integrin β-chain family, and *BDKRB1*, involved in regulation of actin cytoskeleton.

**Table 2:** Differentially expressed genes selected for qPCR validation of microarray results.

	Gene symbol	Function	Fold Change	pV
Upregulated	CLDN1	A component of tight junctions	1.68	0.00021
	ITGB8	Membrane protein involved in cell-cell/extracellular matrix interaction	1.64	0.01039
	BDKRB1	Regulation of actin cytoskeleton	1.58	0.03469
Downregulated	HAPLN1	Cell adhesion, stability of extracellular matrix	-1.77	0.00627
	SCUBE3	Regulates EMT via TGFBRII	-1.66	0.00002
	LMOD1	Actin binding	-1.56	0.01210
	ACTA2	Smooth muscle actin, mesenchymal marker	-1.56	0.00688

### Effect of ALK5 inhibition on the AKT-FOXO1 pathway

**Fig 5:**
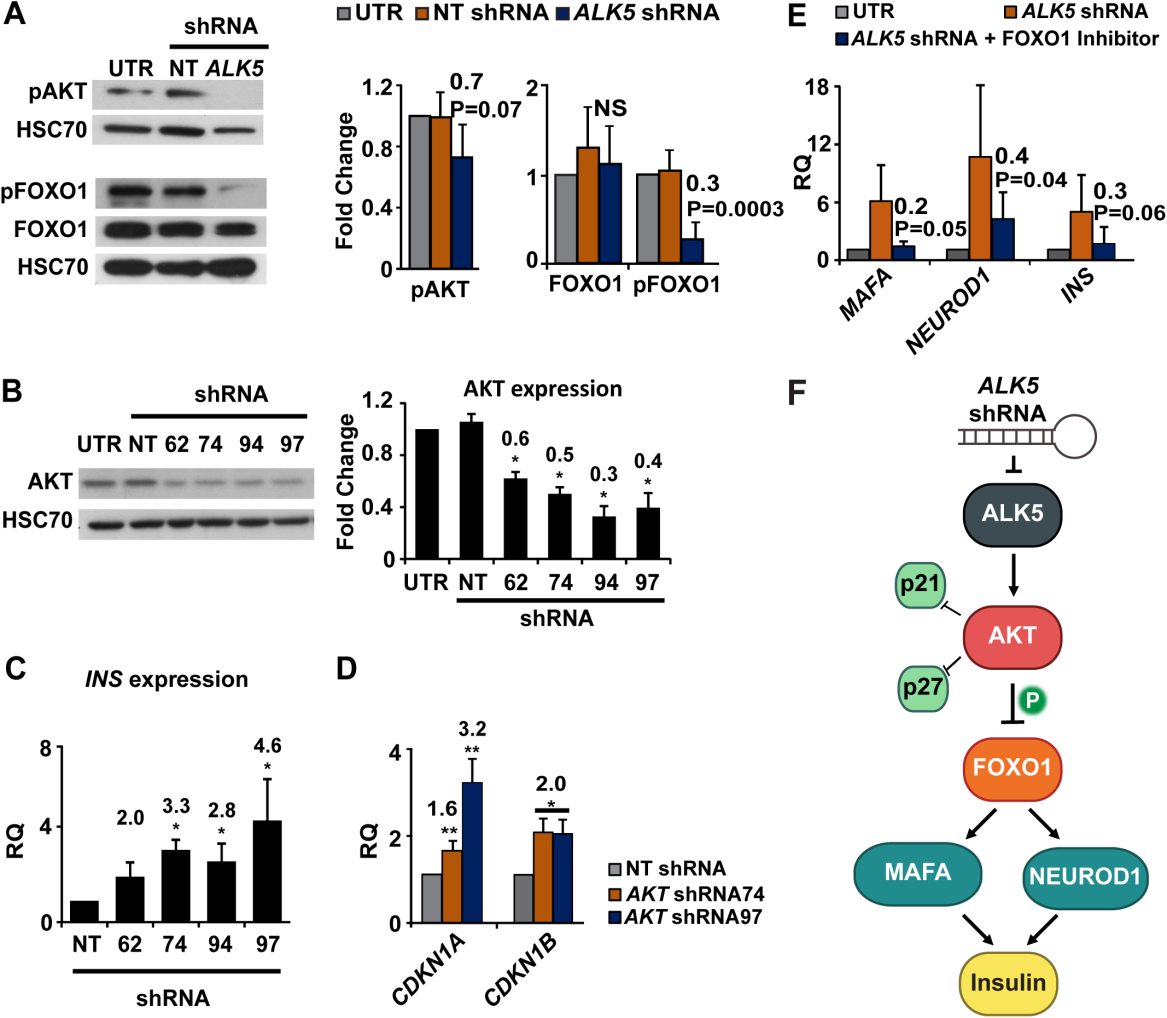
ALK5 shRNA inhibits AKT and activates FOXO1. A, Immunoblotting analysis of phosphorylated AKT, phosphorylated FOXO1, and total FOXO1 proteins in expanded islet cells infected at passage 5 with *ALK5* or NT shRNA viruses and analyzed 6 days later. Values are mean±SE (n = 4–5 donors) relative to UTR and normalized to HSC70. Fold change and P values are relative to NT shRNA. NS, not significant. B, Immunoblotting analysis of AKT in expanded islet cells infected at passage 5–6 with four *AKT* shRNA sequences and analyzed 6 days later. Values are mean±SE (n = 4–5 donors) and normalized to HSC70. *P≤ 0.05. Fold change and P values shown on top of bars are relative to NT shRNA. C, qPCR analysis of RNA extracted from expanded islet cells infected at passage 5–6 with *AKT* or NT shRNA viruses and analyzed 6 days later. Values are mean±SE (n = 4–5 donors) relative to NT shRNA and normalized to *RPLPO* and *TBP*. *P≤ 0.05. D, qPCR analysis of transcripts encoding cell cycle inhibitors in RNA extracted from expanded islets cells infected at passage 5 with *AKT* or NT shRNA viruses and analyzed 6 days later. Values are mean±SE (n = 4 donors) relative to NT shRNA and normalized to *RPLPO* and *TBP*. *P≤ 0.05, **P≤0.01. E, qPCR analysis of RNA extracted from expanded islets cells infected at passage 5 with *ALK5* shRNA virus and treated with FOXO1 inhibitor, and analyzed 6 days later. Values are mean±SE (n = 3 donors) relative to UTR and normalized to *RPLPO* and *TBP*. Fold change and P values are relative to *ALK5* shRNA. F, Scheme of suggested mechanism for induction of insulin expression and growth arrest by ALK5 downregulation.

To explore a possible mechanism involved in the effect of ALK5 inhibition on insulin expression and growth arrest, we examined the effects of ALK5 shRNA on the AKT- forkhead box protein O1 (FOXO1) pathway in expanded islet cells. ALK5 has been reported to be an upstream activator of AKT [29–31]. AKT phosphorylates FOXO1 and renders it inactive [32–35]. Both AKT and FOXO1 have been reported to inhibit cell proliferation by downregulation of the cells cycle inhibitors *CDKN1A* and *CDKN1B* [36, 37]. FOXO1 induces insulin gene expression by upregulation of NEUROD1 and MAFA [38], which are key regulators of insulin gene transcription [39–43]. We have documented an increase in the levels of phosphorylated (active) AKT (p-AKT) in cultured human islet cells [44]. Immunoblotting analysis of expanded islet cells treated with *ALK5* shRNA showed a 30% decrease in p-AKT, and a 70% decrease in phosphorylated (inactive) FOXO1, while no significant change was observed in total FOXO1 levels (Fig 5A). Treatment of expanded islet cells with *AKT* shRNA (Fig 5B) resulted in upregulation of *INS* transcripts (Fig 5C), as well as *CDKN1A* and *CDKN1B* transcripts (Fig 5D), encoding the cell cycle inhibitors p21 and p27, respectively. Blocking FOXO1 activity using a small molecule inhibitor lead to an opposite effect of that of *AKT* inhibition. qPCR analysis of RNA extracted from expanded islet cells treated with *ALK5* shRNA and FOXO1 inhibitor revealed a 60–80% lower activation of *MAFA*, *NEUROD1*, and *INS*, compared with cells treated with *ALK5* shRNA alone (Fig 5E). Taken together, these findings suggest a possible mechanism linking ALK5 downregulation, insulin gene expression, and growth arrest during islet cell redifferentiation (Fig 5F).

### Synergy between ALK5 inhibition and RC treatment on islet cell redifferentiation

We have previously reported that expanded islet cells can be redifferentiated by treatment with a combination of soluble factors in serum-free medium, termed Redifferentiation Cocktail (RC) [25]. Immunostaining analysis of expanded islet cells treated with RC revealed a shift of SMAD2/3 from the nucleus to the cytoplasm, and a shift of SMAD1/5/8 into the nucleus (S5 Fig), suggesting involvement of the TGFβ pathway in MET induced by RC in these cells. ALK5 inhibition synergized with RC treatment in upregulation of transcripts encoding insulin, IAPP, and β-cell transcription factors (S5 Fig), as well as in inducing a 2.6-fold increase in the number of C-peptide-positive cells (S5C Fig), compared with cells treated with RC and NT shRNA. Virtually all C-peptide^+^ cells generated by the combined treatment co-stained for NKX2.2 and PDX1 (S5 Fig). qPCR analysis of dedifferentiated expanded islet cells revealed a 10^4^-fold decrease of insulin transcripts, compared to isolated islets [1]. The combined ALK5 shRNA and RC treatment induced a 635-fold upregulation of insulin mRNA transcripts, bringing these cells closer to a β-cell phenotype. Considering that the fraction of C-peptide^+^ cells in the cell population following the combined *ALK5* shRNA-RC treatment was 8.6%, this expression level is estimated at about 74% of *INS* mRNA of normal human islets.

## Discussion

Our findings document the activation of the TGFβ pathway in expanded human BCD cells, and demonstrate that a 40% inhibition of ALK5 expression is sufficient for induction of BCD cell redifferentiation, as manifested in activation of β-cell gene expression, growth arrest, and MET. These effects were reproducible in cells derived from multiple human donors. Treatment with *ALK5* shRNA alone restored C-peptide expression, as well as glucose responsiveness. *ALK5* inhibition also potentiated RC-induced redifferentiation, as judged by a 10-fold increase in expression of *INS* transcripts, up to about 74% of their level in normal human islets, and a 2.6-fold increase in the number of C-peptide^+^ cells, compared with cells treated with RC alone. The redifferentiation of 8.6% of total cells induced by the combined treatment, as judged by immunostaining for C-peptide, is somewhat higher than the redifferentiation we previously reported with other treatments [27, 45]

Our findings suggest the involvement of AKT and its downstream effector FOXO1 in mediating the effects of TGFβ pathway inhibition on redifferentiation and growth arrest of BCD cells. We have recently reported the role of AKT and FOXO1 in mediating the effects of another pathway implicated in EMT in BCD cells, the WNT pathway [27]. In addition to AKT-FOXO1, other elements may mediate the effects of TGFβ pathway in BCD cells. The TGFβ pathway has been reported to affect insulin expression via a repressive action of SMAD3. SMAD3 negatively regulates MAFA-dependent transactivation in mouse and human cells by directly interacting with MAFA protein [46]. Furthermore, SMAD3 has been shown to occupy the *Ins* promoter in β-cell lines and suppress insulin content, whereas SMAD3 small interfering RNAs relieved *Ins* transcriptional repression [47]. The induction of MET as a result of TGFβ pathway inhibition is likely mediated through ZEB1, a key transcription factor which suppresses *CDH1* expression, and activates expression of mesenchymal markers [6]. We have recently shown that inhibition of ZEB1 expression in BCD cells results in MET, as well as in redifferentiation and growth arrest, which are mediated by miR-200c [45].

Our findings demonstrate the key role of the TGFβ pathway in BCD cell dedifferentiation, and suggest that TGFβ pathway inhibition may contribute to protocols of BCD cell redifferentiation, as part of a therapeutic approach to diabetes based on *in-vitro* expansion of islets from a single human donor for transplantation into multiple recipients. This prospect will require the use of effective small molecule inhibitors of the TGFβ pathway, as well as ways for protecting the transplanted cells from allograft rejection and recurring autoimmunity. Furthermore, TGFβ pathway blocking may contribute to reversal of β-cell dedifferentiation *in vivo*, which appears to be involved in the pathology of type 2 diabetes [48].

## Supporting Information

S1 Fig Changes in SMAD1/5/8 expression during culture of human islet cells. A, qPCR analysis of RNA extracted from human islets and expanded islet cells at the indicated passages. Values are mean±SE (n = 6 donors) relative to islets (RQ = 1) and normalized to *RPLPO* and *TBP*. NS, Not significant. B, Immunofluorescence analysis of SMAD1/5/8 in expanded islet cells at passage 3. SMAD1/5/8 is localized in the cytoplasm of GFP^+^ BCD cells. Bar = 50 μM. DNA was stained with DAPI.
(TIF)

S2 Fig Inhibition of ALK5 by shRNA. A, Immunoblotting analysis of p-SMAD2 in expanded islet cells from 2 donors infected at passages 5–6 with five *ALK5* or NT shRNA viruses and analyzed 6 days later. B, qPCR analysis of RNA extracted from expanded islet cells infected at passage 5–6 with *ALK5* or NT shRNA viruses and analyzed 6 days later. Values are mean±SE of technical triplicates, relative to NT shRNA and normalized to *RPLPO* and *TBP*. C, D, Analysis of ALK5 expression in cells infected at passage 5 with *ALK5* shRNA 9 or NT shRNA viruses and analyzed 6 days later. C, qPCR analysis Values are mean±SE (n = 6 donors) relative to UTR and normalized to *RPLPO* and *TBP*. D, Immunoblotting analysis. Values are mean±SE (n = 5 donors) relative to UTR and normalized to HSC70. Fold change and P value are relative to NT shRNA.
(TIF)

S3 Fig Apoptosis analysis. Expanded islet cells were infected at passage 5 with *ALK5* or NT shRNA viruses and analyzed 6 days later by TUNEL assay.
(TIF)

S4 Fig Effect of ALK5 inhibitor II on insulin gene expression. qPCR analysis of RNA extracted from cells infected at passage 5 with *ALK5* or NT shRNA viruses, or treated with 1 μM ALK5 inhibitor II for 4 days. Values are mean±SE (n = 5 donors) relative to UTR and normalized to *RPLPO* and *TBP*.
(TIF)

S5 Fig Synergy between ALK5 inhibition and RC treatment. A, Immunofluorescence analysis of SMAD2/3 and SMAD1/5/8 in human islet cells expanded to passage 5 and treated with RC for 4 days. DNA was stained with DAPI. Bar = 25 μM. Top: 0% of C-pep^+^ cells showed nuclear SMAD2/3 staining; bottom: 100% of C-pep^+^ cells showed nuclear SMAD1/5/8 staining; based on counting ≥500 cells in each of 4 samples from different donors. B, qPCR analysis of transcripts encoding β-cell proteins in RNA extracted from expanded islet cells infected at passage 5 with *ALK5* or NT shRNA viruses and treated 6 days later with RC for 4 days. Values are mean±SE (n = 3–8 donors) relative to UTR and normalized to *RPLPO* and *TBP*. *P≤ 0.05, **P≤0.01. Fold change and P values shown on top of bars are relative to NT shRNA. C, Quantitation of immunofluorescence analysis of C-peptide in GFP^+^ BCD cells infected at passage 5 with *ALK5* or NT shRNA viruses and treated 6 days later with RC for 4 days. Values are mean±SE (n = 4 donors) relative to RC+NT shRNA, based on counting ≥500 cells in each sample. D, Immunofluorescence analysis of GFP^+^ BCD cells infected at passage 5 with *ALK5* shRNA and treated with RC for 4 days. DNA was stained with blue DAPI. Bar = 25 μM.
(TIF)

S1 Table Primer sequences for qPCR analysis. (DOC)

S2 Table Antibodies for immunofluorescence and immunoblotting analyses. (DOC)
