# Supplementary figures and images for: TGFβ Pathway Inhibition Redifferentiates Human Pancreatic Islet β Cells Expanded In Vitro
Source: PLoS One. 2015 Sep 29;10(9):e0139168. doi: 10.1371/journal.pone.0139168 (PMC4587799; doi:10.1371/journal.pone.0139168)

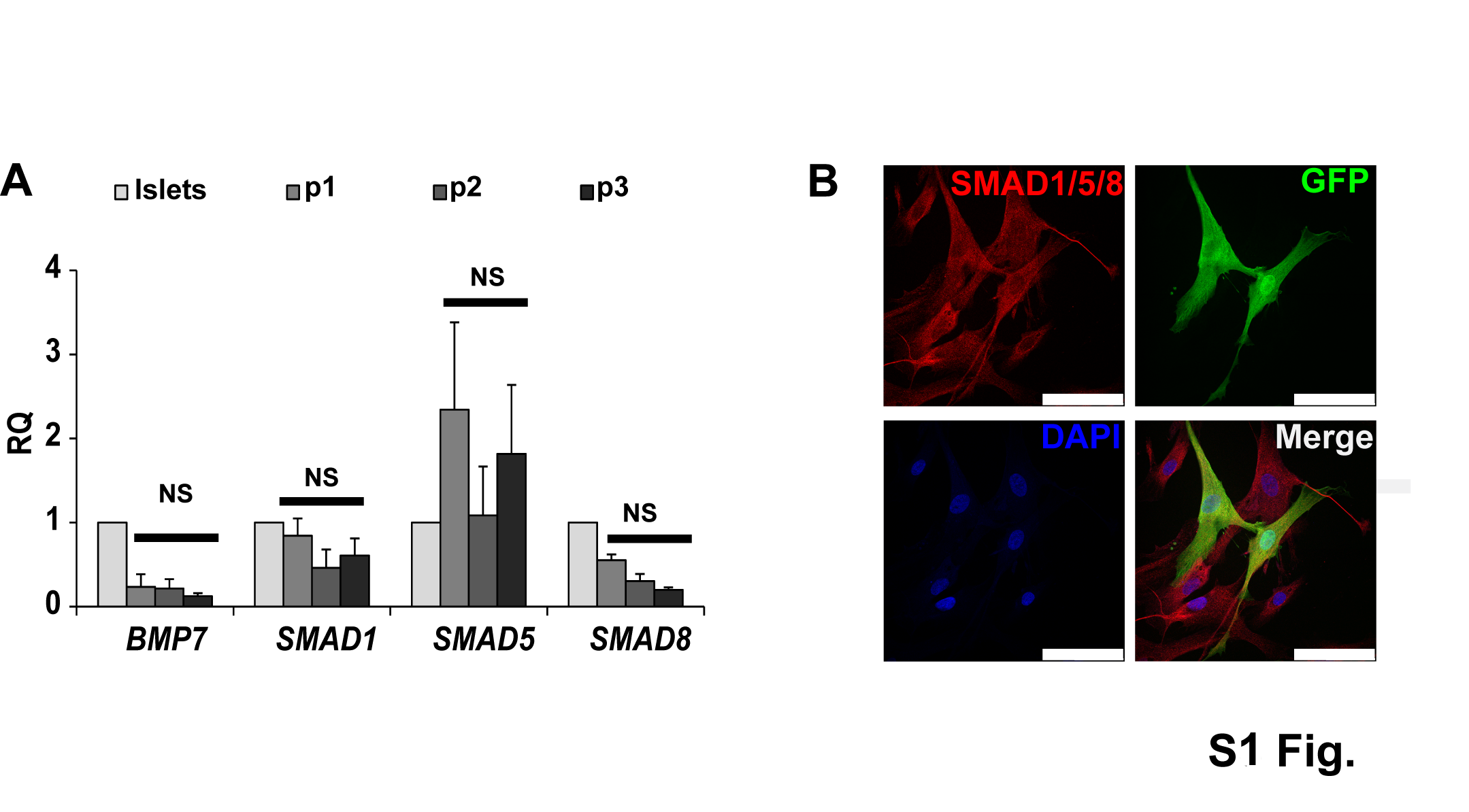

Supplement: S1 Fig — A, qPCR analysis of RNA extracted from human islets and expanded islet cells at the indicated passages. Values are mean±SE (n = 6 donors) relative to islets (RQ = 1) and normalized to RPLPO and TBP. NS, Not significant. B, Immunofluorescence analysis of SMAD1/5/8 in expanded islet cells at passage 3. SMAD1/5/8 is localized in the cytoplasm of GFP+ BCD cells. Bar = 50 μM. DNA was stained with DAPI. (TIF) [file pone.0139168.s001.tif]

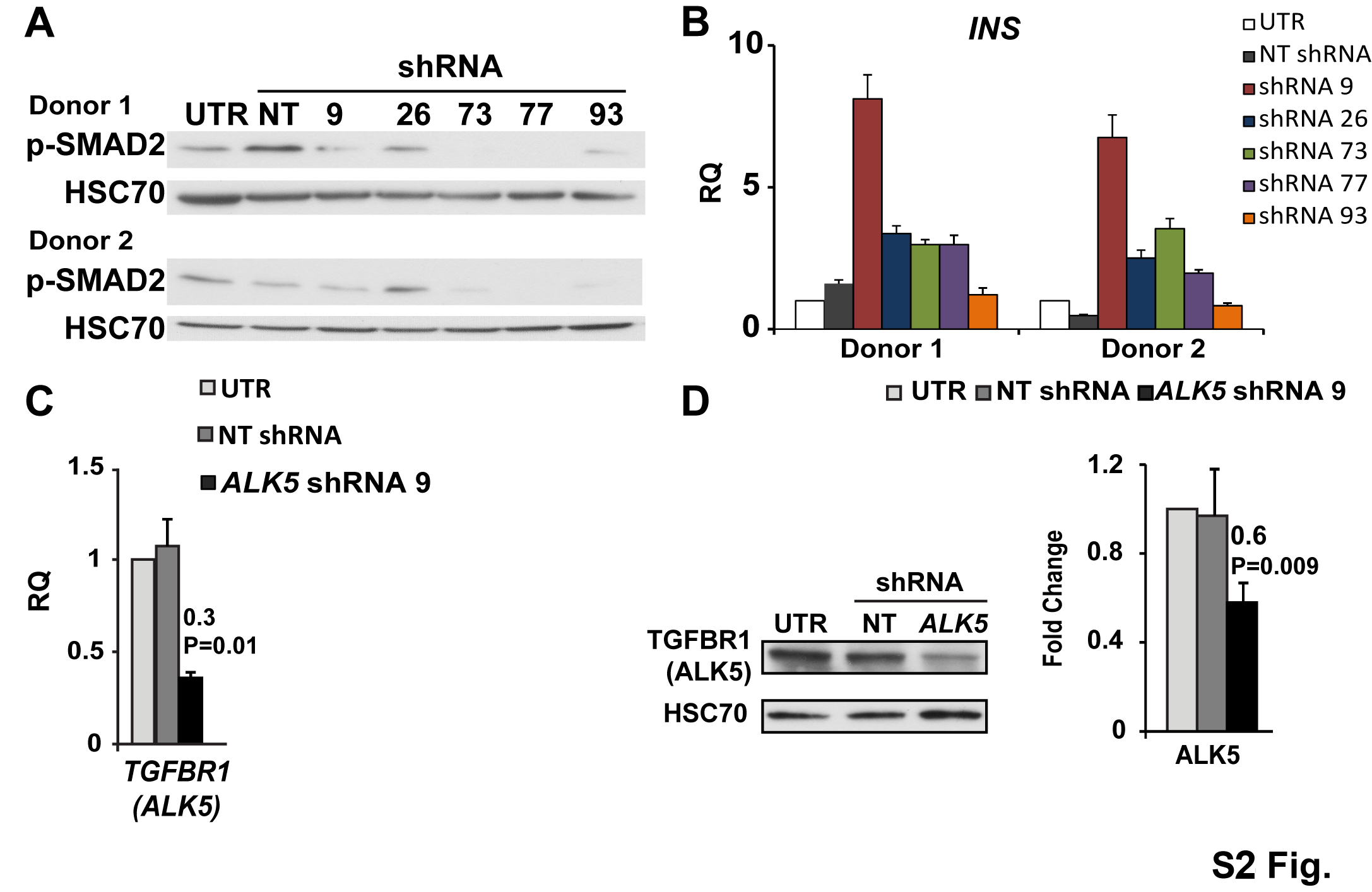

Supplement: S2 Fig — A, Immunoblotting analysis of p-SMAD2 in expanded islet cells from 2 donors infected at passages 5–6 with five ALK5 or NT shRNA viruses and analyzed 6 days later. B, qPCR analysis of RNA extracted from expanded islet cells infected at passage 5–6 with ALK5 or NT shRNA viruses and analyzed 6 days later. Values are mean±SE of technical triplicates, relative to NT shRNA and normalized to RPLPO and TBP. C, D, Analysis of ALK5 expression in cells infected at passage 5 with ALK5 shRNA 9 or NT shRNA viruses and analyzed 6 days later. C, qPCR analysis Values are mean±SE (n = 6 donors) relative to UTR and normalized to RPLPO and TBP. D, Immunoblotting analysis. Values are mean±SE (n = 5 donors) relative to UTR and normalized to HSC70. Fold change and P value are relative to NT shRNA. (TIF) [file pone.0139168.s002.tif]

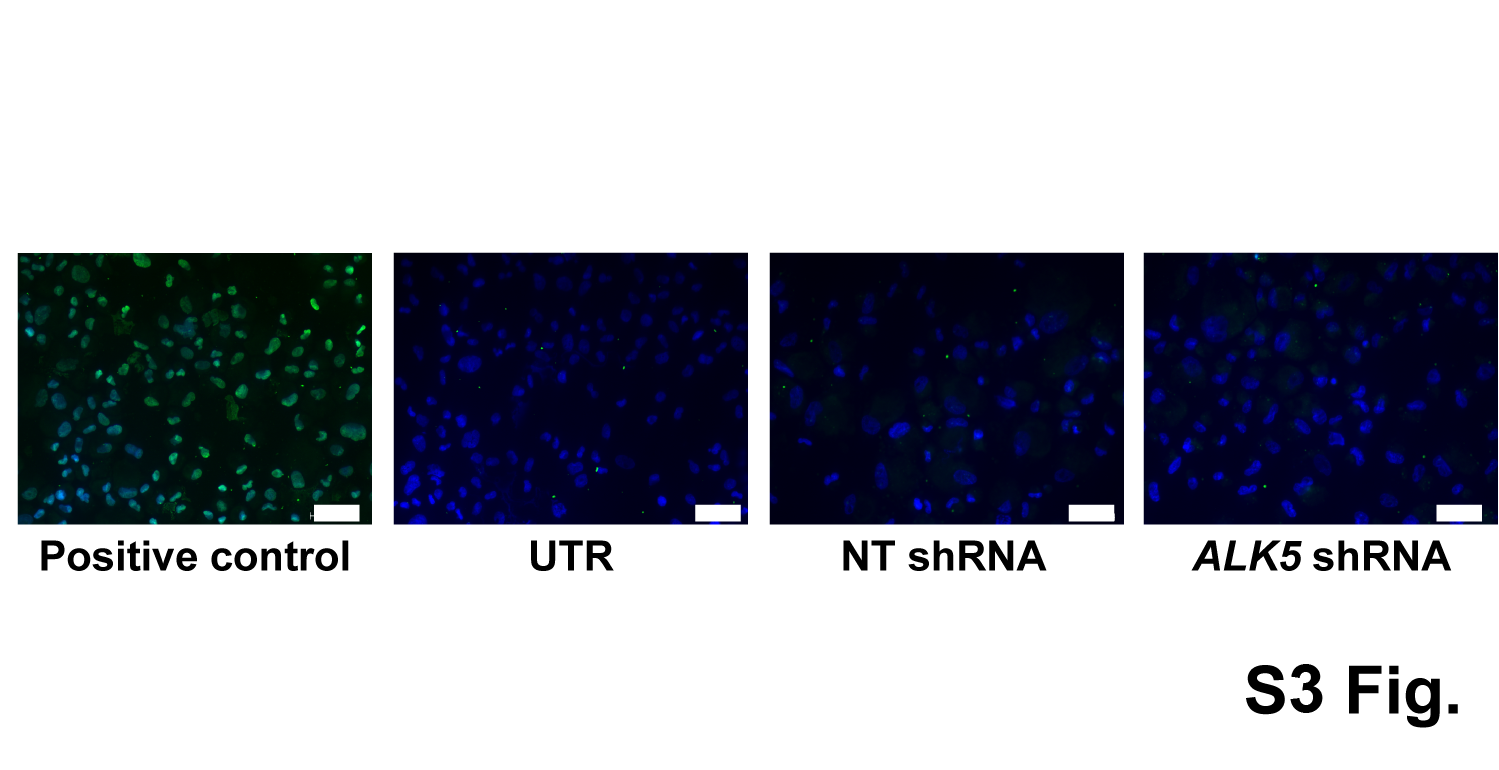

Supplement: S3 Fig — Expanded islet cells were infected at passage 5 with ALK5 or NT shRNA viruses and analyzed 6 days later by TUNEL assay. (TIF) [file pone.0139168.s003.tif]

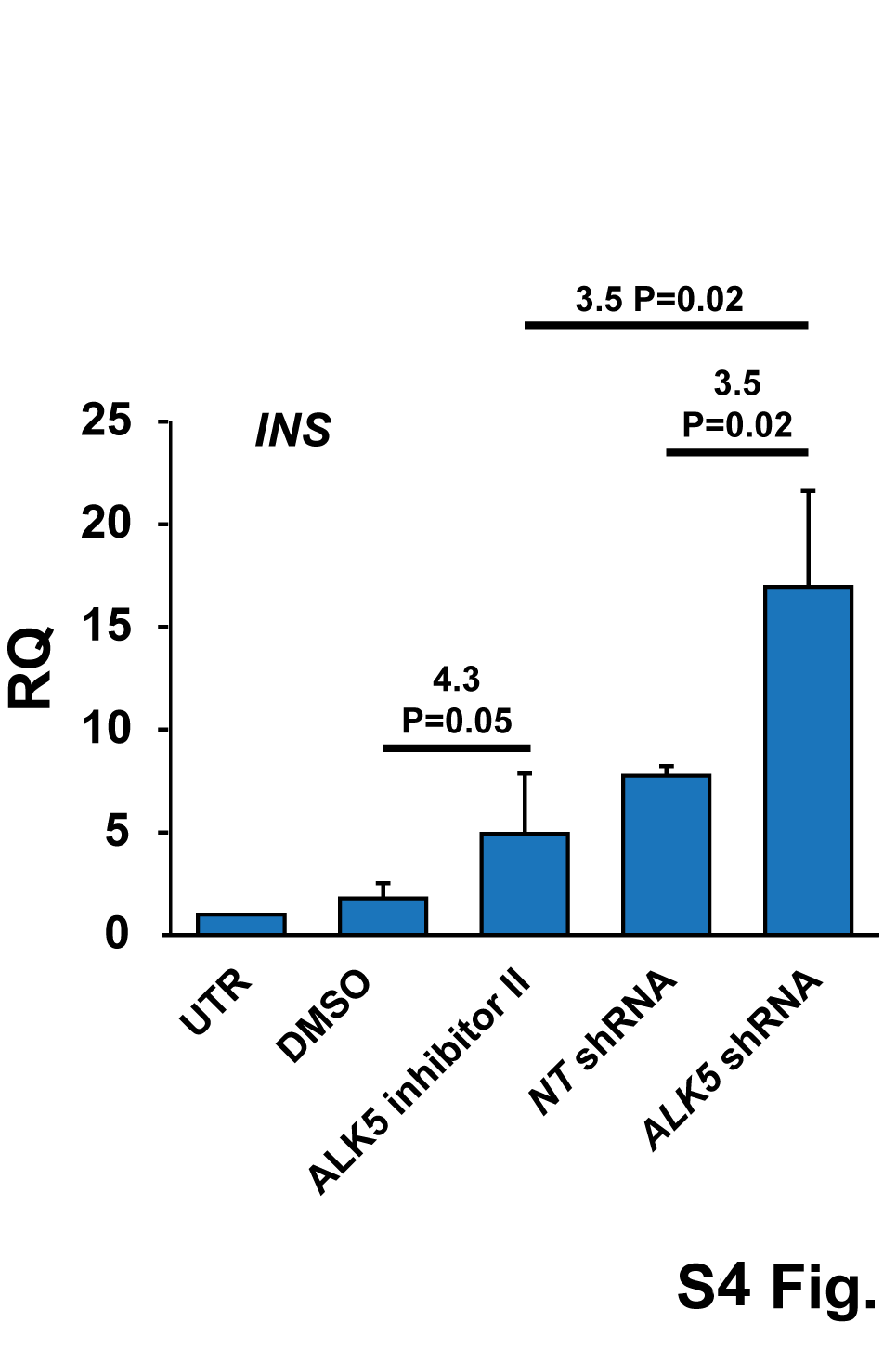

Supplement: S4 Fig — qPCR analysis of RNA extracted from cells infected at passage 5 with ALK5 or NT shRNA viruses, or treated with 1 μM ALK5 inhibitor II for 4 days. Values are mean±SE (n = 5 donors) relative to UTR and normalized to RPLPO and TBP. (TIF) [file pone.0139168.s004.tif]

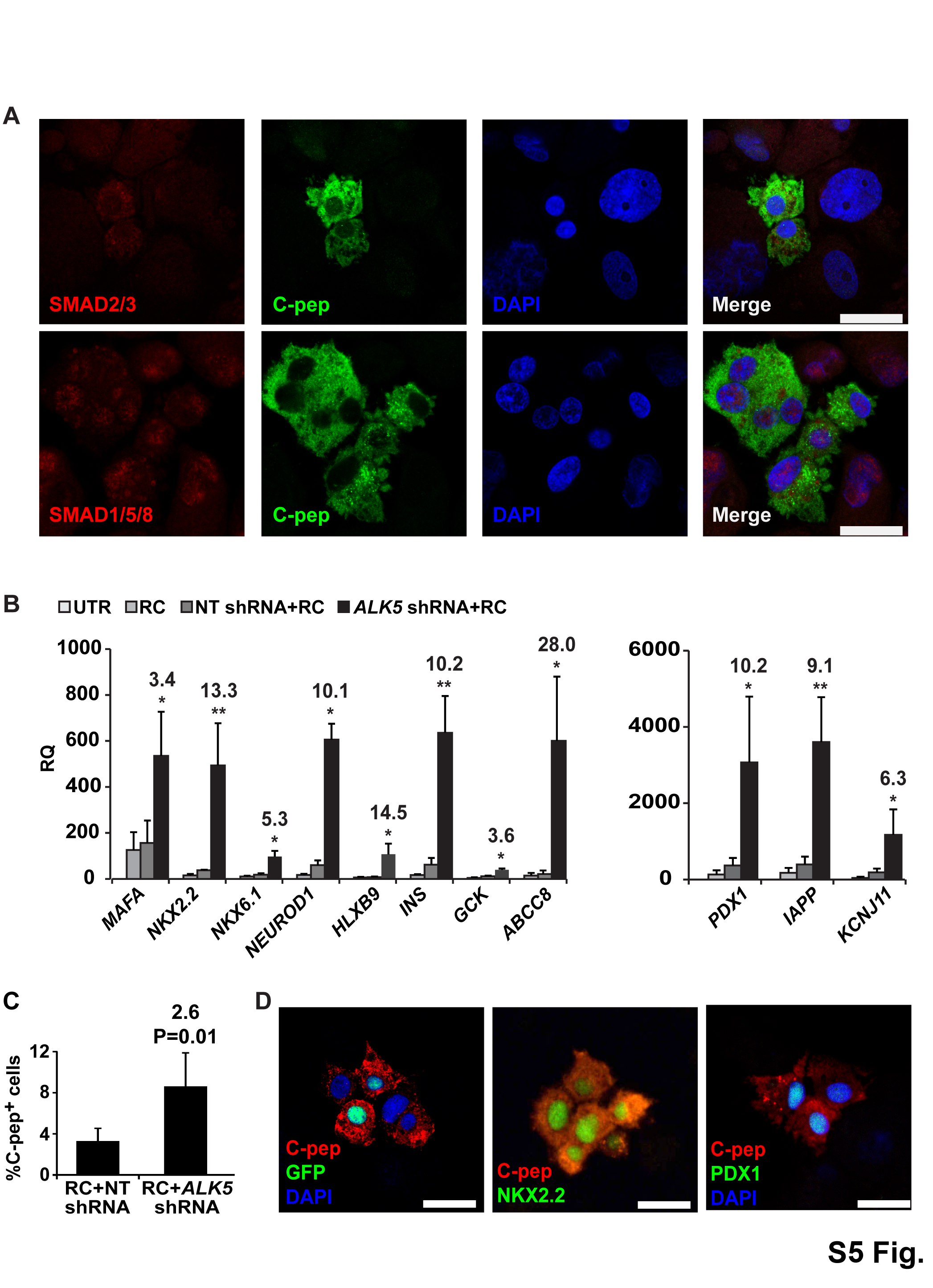

Supplement: S5 Fig — A, Immunofluorescence analysis of SMAD2/3 and SMAD1/5/8 in human islet cells expanded to passage 5 and treated with RC for 4 days. DNA was stained with DAPI. Bar = 25 μM. Top: 0% of C-pep+ cells showed nuclear SMAD2/3 staining; bottom: 100% of C-pep+ cells showed nuclear SMAD1/5/8 staining; based on counting ≥500 cells in each of 4 samples from different donors. B, qPCR analysis of transcripts encoding β-cell proteins in RNA extracted from expanded islet cells infected at passage 5 with ALK5 or NT shRNA viruses and treated 6 days later with RC for 4 days. Values are mean±SE (n = 3–8 donors) relative to UTR and normalized to RPLPO and TBP. *P≤ 0.05, **P≤0.01. Fold change and P values shown on top of bars are relative to NT shRNA. C, Quantitation of immunofluorescence analysis of C-peptide in GFP+ BCD cells infected at passage 5 with ALK5 or NT shRNA viruses and treated 6 days later with RC for 4 days. Values are mean±SE (n = 4 donors) relative to RC+NT shRNA, based on counting ≥500 cells in each sample. D, Immunofluorescence analysis of GFP+ BCD cells infected at passage 5 with ALK5 shRNA and treated with RC for 4 days. DNA was stained with blue DAPI. Bar = 25 μM. (TIF) [file pone.0139168.s005.tif]
