# Supplementary material for: TGFβ Pathway Inhibition Redifferentiates Human Pancreatic Islet β Cells Expanded In Vitro
Source: PLoS One. 2015 Sep 29;10(9):e0139168. doi: 10.1371/journal.pone.0139168 (PMC4587799; doi:10.1371/journal.pone.0139168)
Supplement: S1 Table — (DOC) [file pone.0139168.s006.doc]

**S1 Table. Primer sequences for qPCR analysis**

| **Gene Symbol** | **Sense Primer** | **Antisense Primer** |
| --- | --- | --- |
| *ABCC8* | gcactttccgcatcttgg | aaaccccgagaaattgtgtct |
| *ACTA2* | gctttcagcttccctgaaca | ggagctgcttcacaggattc |
| *BDKRB1* | aagtggctcccacaaaagc | ttggccccttcactattgg |
| *BMP7* | gcgtttatcaggtgctcca | ccagagggtacggctgtc |
| *CDH1* | gccgagagctacacgttca | gaccggtgcaatcttcaaa |
| *CDH2* | ctccatgtgccggatagc | cgatttcaccagaagcctctac |
| *CDKN1A* | ccgaagtcagttccttgtgg | catgggttctgacggacat |
| *CDKN1B* | tttgacttgcatgaagagaagc | agctgtctctgaaagggacatt |
| *CDKN1C* | ctcctttccccttcttctcg | tccatcgtggatgtgctg |
| *CLDN1* | cctatgaccccagtcaatgc | acagcaaagtagggcacctc |
| *GCG* | gtacaaggcagctggcaac | tgggaagctgagaatgatctg |
| *GCK* | gcagatgctggacgacag | tcctgcagctggaactctg |
| *HAPLN1* | cctggatttcaggacaagtga | tccagagtatagttgtctgaaagatga |
| *HLXB9* | tgcctaagatgcccgactt | agctgctggctggtgaag |
| *IAPP* | ttaccaaattgtagaggctttcg | ccctgcctctatacactcactacc |
| *INS* | aggcttcttctacacacccaag | cacaatgccacgcttctg |
| *ITGB8* | tttgcagcatcttacatgtcttg | tgtttttcacagcactgattgtt |
| *KCNJ11* | tgtgtcaccagcatccactc | cacttggacctcaatggagaa |
| *LMOD1* | ggaagatgggagacaaagtcc | actgaagcagtttgggcact |
| *MAFA* | agcgagaagtgccaactcc | ttgtacaggtcccgctcttt |
| *NEUROD1* | ctgctcaggacctactaacaacaa | gtccagcttggaggacctt |
| *NKX6.1* | cgttggggatgacagagagt | cgagtcctgcttcttcttgg |
| *PDX1* | cacatccctgccctcctac | gaagagccggcttctctaaac |
| *PLIN2* | cctgagctcacagcagtaaca | gtttctaggtaaaggcagtgtatgc |
| *PPY* | tctagtgcccatttactctggac | gcaggtggacaggagcag |
| *RPLPO* | tctacaaccctgaagtgcttgat | caatctgcagacagacactgg |
| *SCUBE3* | tcaaggagggccactgtaaa | aaatgaccatttcccttgtttg |
| *SMAD1* | tgtgtactatacgtatgagctttgtga | taacatcctggcggtggta |
| *SMAD2* | gcttctctgaacaaaccaggtc | atgtggcaatccttttcgat |
| *SMAD5* | aggcgacatattggaaaagg | tgaggcattccgcatacac |
| *SMAD8* | gcattaacccttaccactaccg | gagctgggggttatattcactg |
| *SST* | accccagactccgtcagttt | acagcagctctgccaagaag |
| *TBP* | cggctgtttaacttcgcttc | cacacgccaagaaacagtga |
| *TGFB2* | ccaaagggtacaatgccaac | cagatgcttctggatttatggtatt |
| *TGFBR1* | aaattgctcgacgatgttcc | cataataaggcagttggtaatcttca |
| *TGFBR2* | caccgcacgttcagaagtc | tggatgggcagtcctattaca |
