# Supplementary material for: TGFβ Pathway Inhibition Redifferentiates Human Pancreatic Islet β Cells Expanded In Vitro
Source: PLoS One. 2015 Sep 29;10(9):e0139168. doi: 10.1371/journal.pone.0139168 (PMC4587799; doi:10.1371/journal.pone.0139168)
Supplement: S2 Table — (DOC) [file pone.0139168.s007.doc]

**S2 Table. Antibodies for immunofluorescence and immunoblotting analyses**

|  | **Antibody** | **Manufacturer** | **Dilution** |
| --- | --- | --- | --- |
| Immunofluorescence antibodies | rat anti-C-peptide | Beta Cell Biology Consortium | 1:1000 |
|  | mouse anti-C-peptide | Biodesign | 1:200 |
|  | mouse anti-PDX1 | R&D Systems | 1:500 |
|  | mouse anti-NKX2.2 | Hybridoma Bank | 1:1000 |
|  | rabbit anti-GFP | Invitrogen | 1:1000 |
|  | mouse anti-GFP | Chemicon | 1:500 |
|  | rabbit anti-SMAD2/3 | Santa Cruz Biotechnology | 1:200 |
|  | mouse anti-SMAD2/3 | BD Transduction Laboratories | 1:100 |
|  | rabbit anti-SMAD1/5/8 | Santa Cruz Biotechnology | 1:200 |
|  | rabbit anti-Ki67 | Zymed | 1:200 |
|  | mouse anti-SMA | Progen | 1:250 |
| Immunoblotting antibodies | mouse anti-HSC70 | Santa Cruz Biotechnology | 1:1000 |
|  | rabbit anti-TGFβ RI (V22) | Santa Cruz Biotechnology | 1:500 |
|  | rabbit anti-pSMAD2 (Ser465/467) | Cell Signaling | 1:1000 |
|  | rabbit anti-pFOXO1 (Ser256) | Cell Signaling | 1:1000 |
|  | rabbit anti-FOXO1 | Cell Signaling | 1:1000 |
|  | rabbit anti-pAKT (Thr308) | Cell Signaling | 1:1000 |
|  | rabbit anti-AKT | Cell Signaling | 1:1000 |
